# Impaired interactions among white‐matter functional networks in antipsychotic‐naive first‐episode schizophrenia

**DOI:** 10.1002/hbm.24801

**Published:** 2019-10-01

**Authors:** Yun‐Shuang Fan, Zehan Li, Xujun Duan, Jinming Xiao, Xiaonan Guo, Shaoqiang Han, Jing Guo, Siqi Yang, Jiao Li, Qian Cui, Wei Liao, Huafu Chen

**Affiliations:** ^1^ The Clinical Hospital of Chengdu Brain Science Institute, MOE Key Laboratory for Neuroinformation University of Electronic Science and Technology of China Chengdu People's Republic of China; ^2^ School of Life Science and Technology, Center for Information in BioMedicine University of Electronic Science and Technology of China Chengdu People's Republic of China

**Keywords:** first‐episode schizophrenia, functional networks, interactions, rsfMRI, white‐matter

## Abstract

Schizophrenia has been conceptualized as a disorder arising from structurally pathological alterations to white‐matter fibers in the brain. However, few studies have focused on white‐matter functional changes in schizophrenia. Considering that converging evidence suggests that white‐matter resting state functional MRI (rsfMRI) signals can effectively depict neuronal activity and psychopathological status, this study examined white‐matter network‐level interactions in antipsychotic‐naive first‐episode schizophrenia (FES) to facilitate the interpretation of the psychiatric pathological mechanisms in schizophrenia. We recruited 42 FES patients (FESs) and 38 healthy controls (HCs), all of whom underwent rsfMRI. We identified 11 white‐matter functional networks, which could be further classified into deep, middle, and superficial layers of networks. We then examined network‐level interactions among these 11 white‐matter functional networks using coefficient Granger causality analysis. We employed group comparisons on the influences among 11 networks using network‐based statistic. Excitatory influences from the middle superior corona radiate network to the superficial orbitofrontal and deep networks were disrupted in FESs compared with HCs. Additionally, an extra failure of suppression within superficial networks (including the frontoparietal network, temporofrontal network, and the orbitofrontal network) was observed in FESs. We additionally recruited an independent cohort (13 FESs and 13 HCs) from another center to examine the replicability of our findings across centers. Similar replication results further verified the white‐matter functional network interaction model of schizophrenia. The novel findings of impaired interactions among white‐matter functional networks in schizophrenia indicate that the pathophysiology of schizophrenia may also lie in white‐matter functional abnormalities.

## 1 INTRODUCTION

Schizophrenia has been conceptualized as a chronically debilitating disorder arising from pathological alterations in white‐matter fibers in the brain (Stephan, Friston, & Frith, 2009; Wernicke, 1906). Specifically, white‐matter structure abnormalities, measured by diffusion tensor imaging (DTI) techniques, have been widely reported in schizophrenia (Ellison‐Wright & Bullmore, 2009; Skudlarski et al., 2013). However, little neuroimaging research has focused on white‐matter functional changes in schizophrenia, meaning that the role of white‐matter function in schizophrenia's pathophysiology remains unknown.

Recently, white‐matter functional activity has been confirmed to have physiological significance by compelling neuroimaging studies. For instance, functional activity in distinct white‐matter tracts has been found to correspond to specific task demands, such as perceptual or motor tasks (Fabri & Polonara, 2013; Fabri, Polonara, Mascioli, Salvolini, & Manzoni, 2011; Gawryluk, Mazerolle, Brewer, Beyea, & D'Arcy, 2011; Gawryluk, Mazerolle, & D'Arcy, 2014). Moreover, during resting state, white‐matter functional signals have been found to be highly homogenous and can effectively depict neuronal activity (Ding et al., 2013, 2016; G.‐J. Ji, Liao, Chen, Zhang, & Wang, 2017; Marussich, Lu, Wen, & Liu, 2017). By using these resting‐state functional signals, white matter can reproducibly be organized into several functional networks, which are associated with certain gray‐matter neurophysiological networks (Peer, Nitzan, Bick, Levin, & Arzy, 2017). Therefore, resting‐state white‐matter functional activity, which is organized in an intrinsic network‐level form, may play an essential role in neurophysiological processes.

More recently, white‐matter functional activity abnormalities have been shown to characterize the pathological status of various psychiatric disorders. For example, resting state white‐matter functional activity is closely related to memory function in Alzheimer's disease (Makedonov, Chen, Masellis, MacIntosh, & Alzheimer's Disease Neuroimaging Initiative, 2016). Moreover, regional‐level and network‐level white‐matter functional activity are both aberrant in Parkinson's disease, and are attributed to patient pathology (G. J. Ji et al., 2018). With regard to schizophrenia, network‐level white‐matter functional abnormalities have also been observed (Jiang et al., 2018). Specifically, the activity of white‐matter networks and network‐coupling is impaired and associated with the disease duration in schizophrenia, thereby indicating progressive abnormalities in the white‐matter functional network. Thus, white‐matter functional networks disturbance might represent a promising marker for neuropsychiatric disorders.

Although a recent study has demonstrated white‐matter network‐coupling abnormalities in schizophrenia (Jiang et al., 2018), the interactions among white‐matter functional networks remain unknown. Among‐network interactions prevailingly underlie brain functional integration, which means that psychological functions are related to interactions among distributed functional networks. Disrupted interactions among gray‐matter functional networks have been demonstrated to play a prominent role in psychopathological processes and to be associated with the symptomatology in schizophrenia (de la Iglesia‐Vaya et al., 2014; Liao et al., 2018; Palaniyappan, Simmonite, White, Liddle, & Liddle, 2013; Pu et al., 2016). For instance, a breakdown was found in the interaction loop between the salience and execution network, which can significantly predict the illness severity of schizophrenia (Palaniyappan et al., 2013). In light of the functional correspondence between white‐matter and gray‐matter networks (Peer et al., 2017), changes in white‐matter functional networks interactions appear to contribute to the psychopathological processes in schizophrenia. Therefore, making explicit the interactions among white‐matter functional networks may facilitate the interpretation of the psychiatric pathological mechanisms in schizophrenia.

To disentangle the effects of underlying disease progression, we recruited 42 antipsychotic‐naive patients with first‐episode schizophrenia (FES) and 38 healthy controls (HCs). Considering increasing concern about reproducibility of neuroscientific findings across different centers (Button et al., 2013), we additionally recruited an independent cohort (including 13 FESs and 13 HCs) from another center to examine the reproducibility of current findings. We examined the interactions among white‐matter functional networks using Granger causality analysis (GCA) on the resting‐state functional MRI (rsfMRI) signals. Unlike delay correlation method which attempts to reveal temporal dependencies among networks (Jafri, Pearlson, Stevens, & Calhoun, 2008), GCA provides estimation of the direct information of their causality by using the multivariate autoregressive model (Liao et al., 2010). Moreover, coefficient GCA (cGCA), a type of GCA method, can yield information concerning about excitatory or inhibitory causal influences among functional networks by using the regression coefficient (Chen et al., 2009). Therefore, it may be an effective method to detect signed and directional interactions among white‐matter functional networks. To further investigate the total influence effect of each white‐matter functional network in the interaction model, we additionally examined the causal outflow/inflow of each network by using a traditionally graph‐theoretic metric, that is, out/in strength. In line with white‐matter functional networks coupling disturbances in schizophrenia documented in a previous study (Jiang et al., 2018), we hypothesized that FESs would exhibit disrupted interactions among white‐matter functional networks, and the disruptions would be associated with the patients' clinical symptoms.

## 2 METHODS

### 2.1 Participants

#### 2.1.1 Primary cohort

A total of 80 right‐handed participants were recruited, including 42 FESs, and 38 sex‐, age‐, and education level‐matched HCs with informed consent (see Table 1 for detailed demographic and clinical information). All examinations were carried out under the guidance of the Declaration of Helsinki. This study was approved by the Ethics Committee of the Second Affiliated Hospital of Xinxiang Medical University, China. Patients were recruited from outpatient treatment settings at the Second Affiliated Hospital of Xinxiang Medical University, a psychiatric hospital with 1,500 beds. Controls were recruited through media advertisements. All the subjects were Chinese Han people living in towns around the hospital, with household earnings around the national average. The diagnosis of schizophrenia was in accordance with the Structured Clinical Interview for DSM‐IV‐TR, patient version (SCID‐I/P), and was confirmed by two trained psychiatrists. All the patients were experiencing first‐episode psychosis with less than 1‐year disease duration. Psychiatric symptomatology of the FESs was evaluated using the Positive and Negative Syndrome Scale (PANSS). Both HCs and their first‐degree relatives were screened using the nonpatient version of the SCID to rule out individuals who presented with any history of neurological disorders or psychiatric illnesses. Participants were excluded if they (a) were <18 years old; (b) had current comorbid substance‐use disorder (daily consumption of substances for at least 1 year); (c) had a history of neurological disorders or family history of hereditary neurological disorders; (d) had gross morphological anomalies as evidenced by brain MRI scans; and (d) had any electronic or metal implants.

**Table 1:** Demographic and clinical characteristics

Characteristic	HCs (*n* = 38)	FESs (*n* = 42)	Group comparisons	
			Statistic values	*p* Values
Sex (male/female)	25/13	27/15	χ^2^ = 0.02	.89^a^
Age (years)	24.76 ± 0.74	24.86 ± 0.74	T_(78)_ = 0.09	.93^b^
Education (years)	11.05 ± 0.47	10.48 ± 0.44	U = 687	.28^c^
Cigarette use (no/yes)	30/8	33/9	χ^2^ = 0.002	.97^a^
Alcohol use (no/yes)	29/9	36/6	χ^2^ = 1.16	.28^a^
Duration of illness (months)	—	8.38 ± 0. 40	—	—
PANSS scores				
Total scores	—	91.90 ± 1.73	—	—
General scores	—	48.14 ± 0.99	—	—
Positive scores	—	25.60 ± 0.58	—	—
Negative scores	—	18.17 ± 0.80	—	—

*Note*: Mean ± SEM.

Abbreviations: HC, healthy controls; FES, antipsychotic‐naive first‐episode schizophrenia patients; PANSS, Positive and Negative Symptom Scale.

a The χ^2^ value for gender distribution was obtained by chi‐square test.

b The T values were obtained by two sample *t* test.

c The U values were obtained by Mann–Whitney tests.

#### 2.1.2 Replication cohort

Considering the methodological novelty of this study and increasing concern about reproducibility of neuroscientific findings (Button et al., 2013), an independent replication cohort was necessary to determine the replicability of current findings. A total of 26 right‐handed participants were recruited (see Table S3 in Supplementary 5 for detailed demographic information), including 13 FESs, and 13 sex‐, age‐, and education level‐matched HCs after providing informed consent according to the guidelines of the First Affiliated Hospital of Chongqing Medical University. Patients were recruited from outpatient treatment settings at the First Affiliated Hospital of Chongqing Medical University, which is a general hospital with 3,200 beds. All the patients were experiencing first‐episode psychosis with less than 1‐year disease duration. Controls were recruited through media advertisements. All the participants were Chinese Han people living in towns around the hospital, with household earnings around the national average. The exclusion criteria were the same for both the primary cohort and the replication cohort. See Supplementary 5 for data acquisition. One FES patient and one HC were excluded for their excessive head motion (see following data preprocessing). Ultimately, 24 subjects took part in further analyses, which were identical with data analyses of the primary cohort described below.

### 2.2 Data acquisition

Imaging data of the primary cohort were collected using a 3.0 Tesla MRI scanner (Siemens Medical Systems, Erlangen, Germany) at the Second Affiliated Hospital of Xinxiang Medical University. Participants were instructed to stay awake with their eyes closed and not to think of anything in particular. T1‐weighted anatomical images were acquired by a three‐dimensional fast spoiled gradient‐echo sequence with the following parameters: TR = 1,900 ms; TE = 2.52 ms; flip angle = 90°; field of view = 250 × 250 mm^2^; matrix = 256 × 256, 176 axial slices; slice thickness = 1 mm, no gap. Resting‐state functional images were acquired using an echo‐planar imaging (EPI) sequence with the following parameters: TR = 2,000 ms; TE = 30 ms; flip angle = 90°; field of view = 220 × 220 mm^2^; matrix = 64 × 64, 33 axial slices; slice thickness = 3 mm, 0.6 mm gap; 240 volumes.

### 2.3 Data Preprocessing

fMRI data of both the primary cohort and the replication cohort were preprocessed using the Data Processing Assistant for resting‐state fMRI software (DPARSF, Advanced Edition, V4.3; http://www.restfmri.net) and Statistical Parametric Mapping toolkits (SPM8; http://www.fil.ion.ucl.ac.uk/spm). T1‐weighted anatomical images were segmented into white‐matter, gray‐matter, and cerebrospinal fluid (CSF) using SPM8's New Segment algorithm and then normalized to the Montreal Neurological Institute template. The first 10 volumes were discarded. The remaining volumes were then slice‐time corrected and motion corrected (cutoff <3 mm or 3°) to the mean functional image, and coregistered with an anatomical image. One FES patient and one HC in the replication cohort were excluded for excessive head movement, and no participant was excluded in the primary cohort. The framewise displacement (FD) was calculated and used to determine motion “spikes” (FD > 1 mm) to further minimize motion effects (J. Guo et al., 2019; X. Guo et al., 2019; Power, Barnes, Snyder, Schlaggar, & Petersen, 2012). The functional images were then detrended to remove linear drifts. Next, nuisance covariates, including 24 head motion parameters (Friston, Williams, Howard, Frackowiak, & Turner, 1996) and the mean CSF signals were regressed out, while the white‐matter and global brain signals (Han et al., 2019) were not regressed out to avoid eliminating signals of interest. Additionally, motion “spikes” were also included as separate regressors to effectively censored the data at the spike without further changes to correlation values (Jiang et al., 2018; R. Li et al., 2018). A 0.01–0.15 Hz temporal band‐pass filter was applied to reduce nonneuronal contributions to BOLD fluctuations. Next, white‐matter functional images were spatially smoothed. Specifically, the T1 segmentation image for each participant was coregistered to the functional space for identifying the white‐matter mask (using a threshold of 0.5 as a previous study suggested) (Peer et al., 2017). The functional images were then smoothed (4 mm full‐width half‐maximum) on the mask. The smoothed white‐matter functional images were used for further analyses. Finally, the images were normalized to the standard EPI template and resampling to 3 mm^3^ cubic voxels.

### 2.4 White‐matter functional networks clustering

The analysis codes were adapted from the online codes published by Peer and colleagues (Peer et al., 2017), and the analysis steps are briefly described here. First, the unified group‐level white‐matter masks were obtained using the T1‐weighted anatomical image segmentation results. Voxels with a percentage of participants >60% were identified as the group‐level white‐matter mask (Peer et al., 2017). The subcortical areas (based on the Harvard‐Oxford Atlas) were removed from the white‐matter mask. Next, the white‐matter functional networks were identified by a clustering approach based on the Pearson's correlation matrices between white‐matter voxels that group masks restricted. K‐means clustering (distance metric‐correlation, 10 replicates) was employed on the averaged correlation matrices across groups. The numbers of clusters ranged from 2 to 22. Finally, by assessing the stability of the number of clusters using an averaged Dice's coefficient (the threshold was set at 0.85), the most stable and detailed white‐matter functional network masks were obtained. (See Supplementary 1 for further details concerning the steps taken in white‐matter functional networks clustering.)

In the primary cohort, according to the Dice's coefficient of each number of clusters, the most stable and detailed white‐matter functional network number was 11 (see Figure S1 in Supplementary 1). Similar to the previous study (Peer et al., 2017), each white‐matter functional network was compared with 20 major white‐matter fiber tracts based on the JHU white‐matter tractography atlas (Hua et al., 2008) to measure the network‐tract correspondence. Moreover, as suggested by Peer et al., these networks were further compared with 48 white‐matter tracts defined by the ICBM DTI workgroup to estimate more accurate correspondence. The symmetrical, interlaced pattern of white‐matter functional networks could be divided into three layers (superficial, middle, and deep). (Detailed network information is shown in Table 2.)

**Table 2:** White‐matter functional networks

Number	White‐matter functional network	Network‐tract correspondence	Layer
1	Deep	Superior longitudinal fasciculus system	Deep
2	Anterior corona radiate	Anterior corona radiata and uncinate fasciculus	Middle
3	Superior corona radiate	Superior corona radiata and superior longitudinal fasciculus	Middle
4	Posterior callosum	Body of corpus callosum and corticospinal tracts	Middle
5	Cerebellar	Inferior corticospinal and posterior cerebellar tracts	Superficial
6	Pre/post‐central	Superior longitudinal fasciculus and cingulum tracts	Superficial
7	Temporofrontal	Corticospinal tracts and anterior thalamic radiation	Superficial
8	Occipital	Forceps major system	Superficial
9	Orbitofrontal	Forceps minor system and anterior thalamic radiation	Superficial
10	Superior temporal	Uncinate and middle temporal lobe tracts	Superficial
11	Frontoparietal	Cingulum and associated tracts	Superficial

*Note*: The network‐tract correspondences were estimated based on the overlap between white‐matter functional networks and two white‐matter tractography atlases provided by Susumu Mori.

In the replication cohort, the most stable and detailed white‐matter functional network number was 11 according to the Dice's coefficient of each number of clusters (see Figure S4 in Supplementary 5), which were also classified into deep, middle, and superficial layers of networks.

### 2.5 Coefficient GCA

The signed and directional influences among 11 white‐matter functional networks were measured using cGCA. For each network, the individual preprocessed resting‐state fMRI time series was extracted by averaging the time series of all voxels within it. The influence strengths among functional networks were evaluated by regression coefficients of the multivariate autoregression model in REST software (V1.8, http://www.restfmri.net) (see Supplementary 2 for details). Each regression coefficient characterizes the signed strength and direction of the relationship between two networks, in line with our previous works (Liao et al., 2018). Positive coefficients denote excitant paths (source activity predicts subsequent increases in target activity), while negative coefficients denote inhibited paths (source predicts subsequent decreases in target). Finally, a directed asymmetric matrix (11 × 11 regression coefficient matrix) was obtained for each participant. The definition of the following cGCA graph‐theoretic metric (Liao et al., 2011) is listed to describe the outflow/inflow influence strengths of each network:

1. Out‐strength: Sum of absolute regression coefficients of certain network where the network is the source variable to significantly predict other networks. It denotes Granger causal afferent connections of each network.
2. In‐strength: Sum of absolute regression coefficients of certain network where the network is the target variable significantly predicted by other networks. It denotes Granger causal efferent connections of each network.

### 2.6 Disturbance of correlation analyses

Considering the relatively low frequency scale of our study, the spread of hemodynamic effects across time may potentially disturb direct correlation at the same time point. Therefore, the seemingly across‐time causality we observed can be simply due to disturbance of correlation. For diluting the effect of direct correlation, the Pearson's correlations among white‐matter functional networks were calculated and added as covariates in group comparison of cGCA in the primary cohort (see Supplementary 2 for further details).

### 2.7 Statistical analyses

In the primary cohort, the within‐group 11 networks influence patterns were examined using one‐sample *t* tests on the influence coefficient matrices across all participants including FESs and HCs. The out/in strengths of each network were calculated within the significant group mask across all subjects. The 11 networks influence differences between FESs and HCs were measured using permutation tests (5,000 times permutations) (Zhang et al., 2011), controlling for sex, age, and education level as confounding variables. Specifically, a permutation distribution of differences was generated by randomly assigning each subject to one of the two groups with the same size as the original FES and HC groups and computing their between‐group differences using two‐sample *t* tests with sex, age, and education level as confounding variables. This procedure was repeated for 5,000 permutations. The *p* value was estimated by calculating the percentage of permutations higher than the actual group difference measured by two‐sample *t* test with the same covariates. The statistical significance level was set at *p* < .05 and network‐based statistic (NBS) adjusted for multiple testing. By using this statistical method, connected subnetworks of edges showing a particular effect of a size larger than which would be expected by chance can be identified (Zalesky, Fornito, & Bullmore, 2010). As the 11 white‐matter networks can be further classified into deep, middle, and superficial layers of networks, for each subject, the out/in strengths of each layer were calculated by summing the absolute out/in strengths of all belonging networks. The out/in strengths of tri‐layer networks were compared between FESs and HCs using two‐sample *t* tests (*p* < .05), false discovery rate (FDR) corrected. The relationships between altered 11 networks influence values and symptom severity (PANSS‐N or PANSS‐P scores) were investigated using Pearson's correlation analyses in FESs.

In the replication cohort, the within‐group networks influence patterns were also examined using one‐sample *t* tests across all participants including FESs and HCs. Group differences on the 11 networks influence between FESs and HCs were measured using two‐sample *t* tests (*p* < .05, FDR corrected), controlling for sex, age, and education level as confounding variables. The out strengths of tri‐layer networks were compared between FESs and HCs using two‐sample *t* tests (*p* < .05, FDR corrected).

### 2.8 Validation analyses

Considering that potential leakage of the gray‐matter fMRI signal may affect the clustering pattern of the white‐matter fMRI signal, two relative stricter group‐level masks (percentages >70 and 80%) were applied to identify white‐matter voxels to validate the consistency of the main results of the primary cohort (see Supplementary 3 for further details).

## 3 RESULTS

### 3.1 Results of the primary cohort

#### 3.1.1 Within‐group networks influence patterns

**Figure 1:**
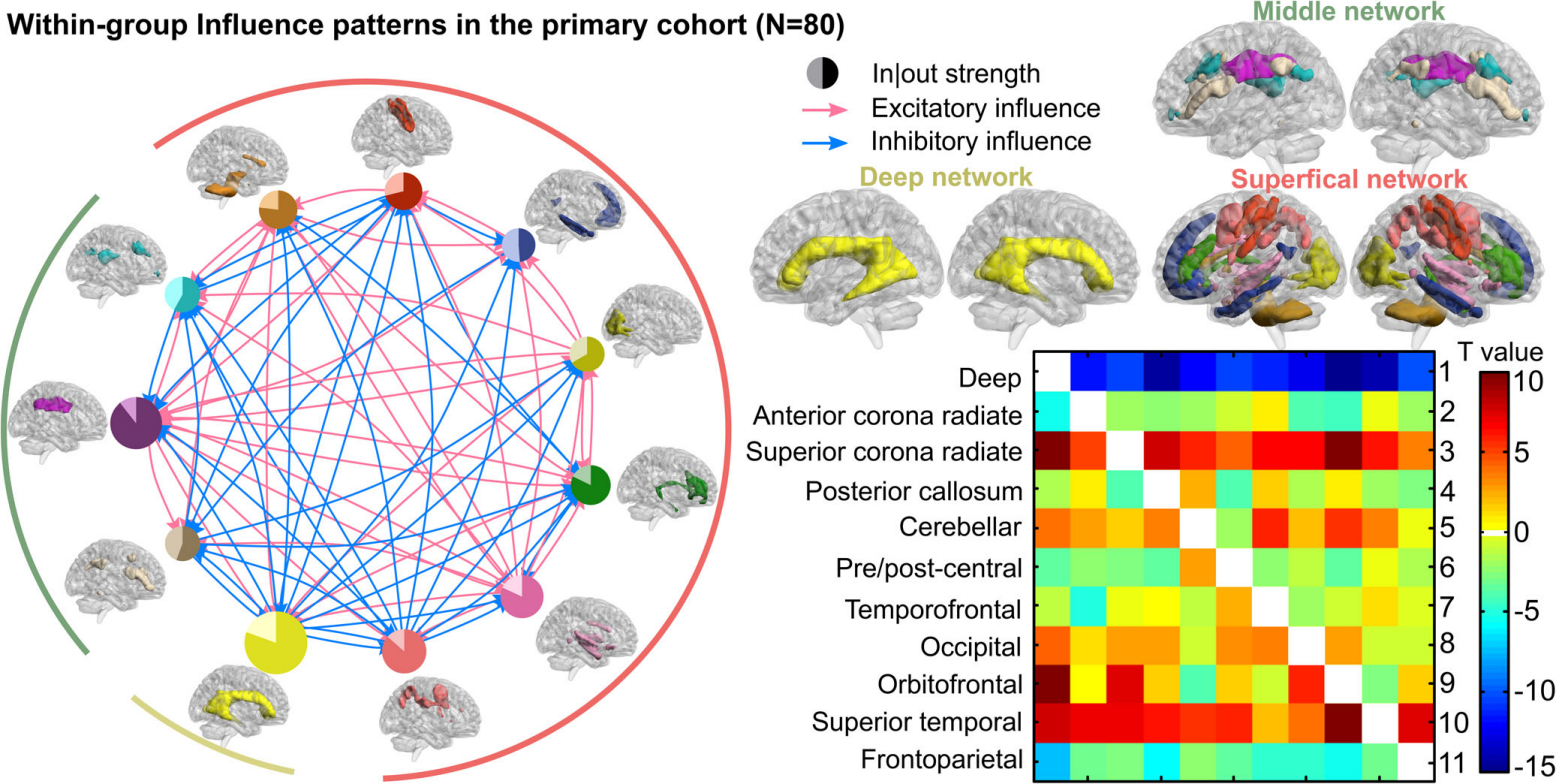
Within‐group white‐matter functional networks influence patterns across all subjects in the primary cohort (*N* = 80) determined by one‐sample *t* test (*p* < .05). Red and blue arrow lines in the left wheel represent significant excitatory influence and inhibitory influence, separately. Positive/negative values in the right T‐value matrix also denote excitatory/inhibitory influence. Excitatory influence represents that source activity predicts subsequent increases in target activity, and inhibitory influence represents source predicts subsequent decrease in target. The circle size of each network on the wheel represents the sum of absolute values of all significant influences whether outflow or inflow this network. In each circle, the light and dark portions refer to inflow and outflow influence strengths, respectively. The 11 networks outside the wheel are presented in clockwise order

The interactions among 11 white‐matter functional networks were evaluated by influence coefficients among networks. The within‐group networks influence patterns (Figure 1) were examined using one‐sample *t* tests (*p* < .05) across all participants (*N* = 80) including FESs and HCs in the primary cohort. In the significant networks influence patterns, a larger total influence strength can be observed in the deep network compared with other networks, partly in accordance with previously reported observations of unique features in deep network (Jiang et al., 2018; Peer et al., 2017).

#### 3.1.2 Between‐group networks influence differences

**Figure 2:**
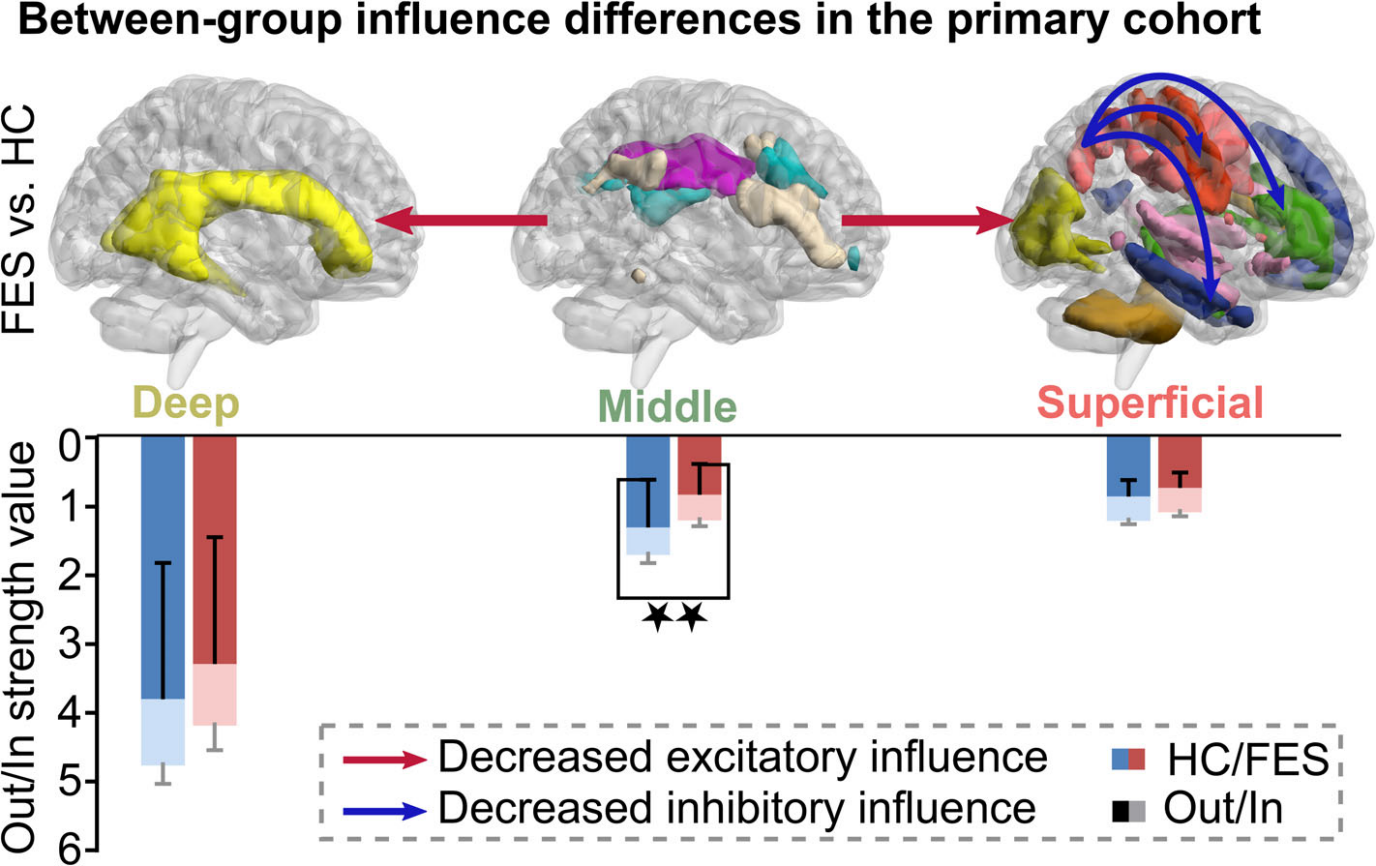
Between‐group white‐matter functional networks influence differences in the primary cohort examined by two‐sample *t* test with 5,000 permutations (*p* < .05, NBS corrected). FESs exhibited significantly decreased excitatory influence from the middle network to the superficial network (*t* = −3.62, *p* < .001) as well as the deep network (*t* = −3.25, *p* = .001) compared to HCs. FESs exhibited additional decreased inhibitory influences within superficial networks. The out strength of the middle networks was decreased in FESs compared with HCs (*t* = −2.90, *p* = .004). FES, antipsychotic‐naive first‐episode schizophrenia patient; HC, healthy control; FDR, false discovery rate. ★★ denotes *p*_FDR‐corrected_ < .05

Differences in the 11 networks influence between FESs and HCs in the primary cohort were measured using two‐sample *t* tests with 5,000 permutations (*p* < .05, NBS corrected; see Figure S3 in Supplementary 4). These differences could be concluded as following tri‐layer network‐level results (Figure 2). Compared with HCs, FESs exhibited decreased excitatory influences from the middle network (i.e., superior corona radiate network) to the superficial network (i.e., orbitofrontal network; *t* = −3.62, *p* = .0007), as well as the deep network (*t* = −3.25, *p* = .001). The out strength of the middle networks was decreased in FESs compared with HCs (*t* = −2.90, *p* = .004, FDR corrected). Altogether, the regulation of middle networks was disrupted in FESs. Additionally, FESs exhibited decreased inhibitory influences within superficial networks including influences from the frontoparietal network to the pre/post‐central network (*t* = −2.94, *p* = .004), temporofrontal network (*t* = −2.98, *p* = .002), and the orbitofrontal network (*t* = −3.04, *p* = .001). No clinical correlation was found between the altered networks influence values and symptom severity in FESs.

### 3.2 Validation results

Both the main results of 70 and 80% group‐level masks were consistent with the main results of 60% masks (see Figure S2 in Supplementary 3). Thus, the main results were still stable even with stricter masks.

### 3.3 Results of the replication cohort

**Figure 3:**
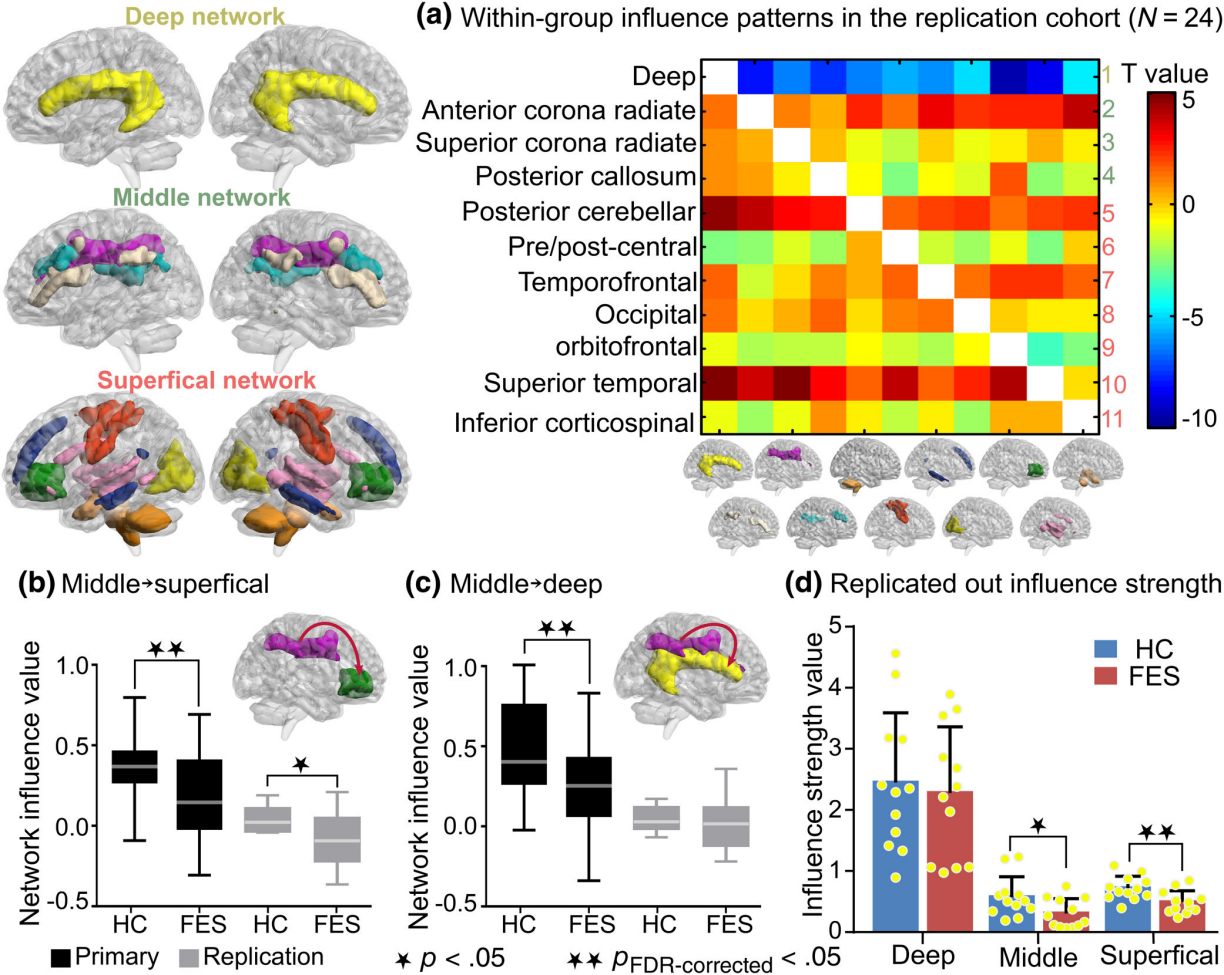
Replications of networks influence patterns and tri‐layer networks influence differences in an independent cohort (*N* = 24). (a) Within‐group influence patterns determined by one‐sample *t* tests across FESs and HCs. Positive and negative values represent excitatory influence and inhibitory influence, separately. (b) In the replication cohort, compared with HCs, the middle￫superficial excitatory influence (*t* = −2.38, *p* = .03) showed significant decrease in FESs, which was similar with the primary cohort. (c) In the replication cohort, no significant decrease on the excitatory influence of middle￫deep network (*t* = −0.65, *p* = .52) was observed in FESs compared to HCs. (d) The out strength of middle network influence was significantly reduced (*t* = −2.25, *p* = .03) in FESs compared to HCs. Additionally, the outflow influence of the superficial network was significantly decreased in FESs (*t* = −2.94, *p* = .008). ★ denotes *p* < .05. ★★ denotes *p*_FDR‐corrected_ < .05. FES, antipsychotic‐naive first‐episode schizophrenia patient; HC, healthy control

Considering the methodological novelty of this study, an independent replication cohort (*N* = 24) was recruited and identically analyzed to examine the reproducibility of current findings. Similarly, 11 white‐matter functional networks were identified, including the deep, anterior corona radiate, superior corona radiate, posterior callosum, posterior cerebellar, pre/post‐central, temporofrontal, occipital, orbitofrontal, superior temporal networks, and the inferior corticospinal network (Figure 3). Notably, the frontoparietal and pre/post‐central networks were merged into a single network, whereas the cerebellar network was subdivided into anterior and posterior subnetworks in the replication results. First and foremost, the within‐group influence patterns (Figure 3a) measured by one‐sample *t* tests across all subjects in the replication cohort were similar to that in the primary cohort. With regard to between‐group differences (two‐sample *t* test, correcting for sex, age, and education level), FESs in the replication cohort exhibited similarly decreased middle→superficial (i.e., superior corona radiate→orbitofrontal) excitatory influence (*t* = −2.38, *p*_unc_ = .03; Figure 3b) compared to HCs. Despite of no significant decrease on the middle→deep excitatory influence (*t* = −0.65, *p* = .52; Figure 3c) in FESs of the replication cohort, the total outflow of the middle network showed a relative decline in FESs compared to HCs (*t* = −2.25, *p*_unc_ = .03; Figure 3d). In addition, the outflow influence of the superficial network was significantly decreased in FESs of the replication cohort (*t* = −2.94, *p* = .008; Figure 3d), indirectly confirmed the disrupted interaction within superficial white‐matter networks. The parsimonious group comparison results in the replication cohort may be largely due to the small sample size. Additional participants should be recruited to further validate the white‐matter network interactions model of FESs. Nevertheless, the replication results were approximately consistent with results of the primary cohort, indicating the replicability of current findings.

## 4 DISCUSSION

In the last decade, the existence of white‐matter signals has been confirmed by compelling evidences. For example, in addition to task‐related activation in distinct white‐matter tracts (Fabri et al., 2011; Fabri & Polonara, 2013; Gawryluk et al., 2011, 2014), the white‐matter signals also exhibit a topological organization during the resting state (G.‐J. Ji et al., 2017; J. Li et al., 2019), indicating that white‐matter signals are more than just noise. Moreover, abnormal white‐matter signals have been reported in various psychiatric disorders, including schizophrenia (G. J. Ji et al., 2018; Jiang et al., 2018; Makedonov et al., 2016). Therefore, the white‐matter signals have a physiological function, and their abnormalities are closely linked to psychopathological status. In view of the network organization pattern of white‐matter suggested by a previous study (Peer et al., 2017), this study examined interactions among 11 white‐matter functional networks consisting of three layers (superficial, middle, and deep) in FESs and HCs. Despite that converging evidences have proved the neurobiological significance of white‐matter signals, the novelty of this field made it necessary to retest current results in another independent sample. However, as we expected, similar retest results confirmed the replicability of white‐matter functional network interaction model of schizophrenia. The present study is the first to investigate the white‐matter functional network interaction model in FESs to advance our understanding of psychiatric pathological mechanisms in schizophrenia.

The neural substrate of functional interactions within white‐matter networks is an issue worthy of discussion. Anatomically, superficial white‐matter tracts connect distant cortical neuron cell bodies that are engaged in different functions, while deep white‐matter tracts are less surrounded by gray matter. With respect to brain function, there was synchronous neural activity within superficial white‐matter networks and cortical gray‐matter networks, whereas hardly no correlation pattern was observed between middle or deep white‐matter networks and any gray‐matter networks (Ding et al., 2018; Peer et al., 2017). In light of the notion that superficial white‐matter tracts and corresponding gray‐matter cortices share a common functional role (Peer et al., 2017), it is reasonable to infer that superficial white‐matter network interactions can be indirectly due to the interplay of cortical gray‐matter network. However, relative to surface white matter, deep white‐matter tracts exhibited more unique features, for example, different spectral profile (Jiang et al., 2018) and hemodynamic response function (M. Li, Newton, Anderson, Ding, & Gore, 2019) from gray matter, suggesting that interactions within middle/deep white‐matter networks subserve specific functions that are less relevant to gray‐matter networks. In conclusion, superficial white‐matter networks may indirectly interplay with each other through gray‐matter networks, while middle and deep white‐matter networks are more likely to communicate directly by axon‐to‐axon interactions.

A generally impaired interactions among white‐matter functional networks was found in the present study, rather than a compensation mechanism on white‐matter networks functional connectivity as suggested by Jiang et al. (2018), that is, middle‐deep white‐matter networks had increased connectivity with superficial networks. This inconsistency may be partly due to the type of patients we recruited. The previous study used patients with chronic schizophrenia, while we used FES patients. These two types of schizophrenia show extensive differences in brain structures (Ellison‐Wright, Glahn, Laird, Thelen, & Bullmore, 2008) and functions (T. Li et al., 2017). Thus, our findings focus on FES patients' functional changes of white‐matter networks. Another possible explanation for the inconsistency in findings relates to the status of antipsychotic medications usage. Patients in the previous study received antipsychotic medications, while our patients were medication‐naive. Therefore, our study was able to explore brain changes derived from disease without the influence of antipsychotic medications (Kahn et al., 2008; Radua et al., 2012). A further potential explanation for the conflict may be the GCA method that we used. The functional connectivity analysis that was previously utilized can only measure the statistical dependence between neuronal activities in distinct networks, but it cannot evaluate the influence that one network exerts over another (Liao et al., 2010, 2011). Therefore, by using the GCA method, the present study explored the pathological alterations of schizophrenia from the perspective of white‐matter functional networks interactions.

The influences from middle white‐matter functional networks to superficial and deep white‐matter functional networks were disrupted in FESs. The middle white‐matter networks were anchored on the corona radiata, which contain reciprocal connecting fibers that link cortex and subcortical bodies (e.g., the basal ganglia; Catani & Thiebaut de Schotten, 2008). Anatomically, the corona radiata first grows and then atrophies with age during normal development (Asato, Terwilliger, Woo, & Luna, 2010; Burzynska et al., 2010), while it abnormally atrophies in schizophrenia (Cui et al., 2011; Walther et al., 2011). By using a novel method, the present study enabled the detection of potential dynamic information transfer through the corona radiate fibers. Our results revealed damaged information export from the middle corona radiate networks in schizophrenia, which additionally support the structural disruption. Specifically, this information export disruption was decomposed into its descending excitatory regulation to deep white‐matter networks and its ascending excitatory regulation to superficial orbitofrontal white‐matter network. According to our inference, the middle corona radiate network should downstream regulate the deep white‐matter network through deep axon‐to‐axon white‐matter tracts, hinting that the path plausibly subserves a communication function (Peer et al., 2017). Combining with the impaired upstream regulation to the superficial orbitofrontal white‐matter network, the damaged information export of the middle networks might explain the cognitive and/or perceptual‐motor deficits in schizophrenia (Carlson, 2014). Altogether, the middle white‐matter networks disengagement in the network interactions model may play a fundamental role in white‐matter dysfunction in schizophrenia.

A particular failure of suppression within the superficial white‐matter functional networks was observed in FESs. Specifically, the regulation from frontoparietal white‐matter network was damaged in FESs, which are coincident with previous gray‐matter findings of a dominant disruption of frontoparietal control network in schizophrenia (Baker et al., 2014). Therefore, dysfunction of frontoparietal network, whether at cortical gray‐matter level or underlying white‐matter level, plays a critical role in the pathology of schizophrenia. Moreover, it is interesting to note that the major surface white‐matter disturbance, which involved in reduced suppression from frontoparietal white‐matter network to temporofrontal and orbitofrontal white‐matter networks, is in line with the dysconnection model of schizophrenia (Friston & Frith, 1995; Stephan et al., 2009). As Friston and Frith suggested, disrupted suppression from frontoparietal gray‐matter network to temporal gray‐matter network might result in a failure to integrate intrinsically generated behavior and extrinsically generated percepts, which could be a sufficient explanation for various symptoms in schizophrenia. More importantly, the correspondence between superficial white‐matter and gray‐matter findings in schizophrenia potentially confirmed our inference that superficial white‐matter network interactions were driven by the interplay of cortical gray‐matter networks. Taken together, the current findings reveal the relevance between functions of cortical gray‐matter networks and superficial white‐matter networks, and further support the dysconnection model of schizophrenia in the aspect of white‐matter function.

We acknowledge several limitations in the present study. Foremost, the sample size was relatively small, which may partly contribute to the failure to find any clinical correlations in patients. Future studies should recruit additional participants to validate the relationship between impaired white‐matter functional networks interactions and schizophrenia symptoms. Second, the parsimonious replication results were underpowered to support the main findings of the present study, which may be due to the small size of the replication cohort. Next, the lack of cognitive function measures was underpowered to support the association between disruption in white‐matter networks regulation and cognitive deficits in schizophrenia. Finally, limited by utilizing a cross‐sectional design, the present study could not uncover the progressive pathological alterations in the white‐matter functions in schizophrenia. Further longitudinal studies that include a medication‐treated patient group are highly recommended.

## 5 CONCLUSIONS

This study sought to elaborate upon the pathological alterations of schizophrenia in terms of white‐matter functional network interactions. The current findings suggest that the middle white‐matter networks disengagement may play a fundamental role in white‐matter dysfunction in schizophrenia. Furthermore, failed suppression within the superficial white‐matter networks in schizophrenia adds additional supports for the dysconnection hypothesis in the aspect of white‐matter function. Overall, the present study uncovered impaired interactions among the white‐matter functional networks in schizophrenia, thereby indicating that the pathophysiology of schizophrenia may also lie in white‐matter functional abnormalities.

## CONFLICT OF INTEREST

The authors declare that they have no conflict of interest.

## Supporting information


**Appendix S1:** Supporting information

## Data Availability

We have published our white‐matter masks and resulting files at https://osf.io/8pvwe.
