# Supplementary material for: Impaired interactions among white‐matter functional networks in antipsychotic‐naive first‐episode schizophrenia
Source: Hum Brain Mapp. 2019 Oct 1;41(1):230–40. doi: 10.1002/hbm.24801 (PMC7267955; doi:10.1002/hbm.24801)
Supplement: Supplementary file 1 — Appendix S1: Supporting information [file HBM-41-230-s001.doc]

**Supplementary Materials**

**Impaired interactions among white-matter functional networks in antipsychotic-naive first-episode schizophrenia**

Running Title: Dysfunction of white-matter networks in schizophrenia

**Yun-Shuang Fan^1,2^, Zehan Li^1,2^, Xujun Duan^1,2^, Jinming Xiao^1,2^, Xiaonan Guo^1,2^, Shaoqiang Han^1,2^, Jing Guo^1,2^, Siqi Yang^1,2^, Jiao Li^1,2^, Qian Cui^1,2^, Wei Liao^1,2*^, Huafu Chen^1,2*^**

^1^ The Clinical Hospital of Chengdu Brain Science Institute, MOE Key Laboratory for Neuroinformation, University of Electronic Science and Technology of China, Chengdu 610054, P.R. China.

^2^ School of Life Science and Technology, Center for Information in BioMedicine, University of Electronic Science and Technology of China, Chengdu 610054, P.R. China.

* Corresponding author:

Huafu Chen, The Clinical Hospital of Chengdu Brain Science Institute, MOE Key Laboratory for Neuroinformation, University of Electronic Science and Technology of China, Chengdu 610054, P.R. China. E-mail: chenhf@uestc.edu.cn (H. Chen). Fax: +86-28-61831273. Tel: +86-28-61831273.

or

Wei Liao, The Clinical Hospital of Chengdu Brain Science Institute, MOE Key Laboratory for Neuroinformation, University of Electronic Science and Technology of China, Chengdu 610054, P.R. China. E-mail: weiliao.wl@gmail.com

**Supplementary 1.**

The unified group-level white-matter masks were obtained using the T1-weighted anatomical image segmentation results. In detailed, individual voxels were first classified into white-matter, gray-matter or CSF based on their maximum probability from segmentation results to obtain white-matter, gray-matter and CSF masks for each subject. Next, these masks were averaged across all the subjects, and the percentage of subjects in which it was classified as white-matter or gray-matter were obtained for each voxel. With regard to white-matter, voxels with a percentage of subjects >60% were identified as the group-level white-matter mask . Additionally, to correctly classified deep brain structures , the subcortical areas (based on the Harvard-Oxford Atlas) were removed from the white-matter mask. Finally, the group-level anatomical white-matter mask was coregistered to the functional space and resampled for restricting functional image processing.

The white-matter functional networks were identified by a clustering approach based on the Pearson's correlation matrices between white-matter voxels. First, to reduce computational complexity, an interchanging grid method was used to subsample the white-matter mask . Specifically, any second voxels along the image rows and columns were taken and shifted by 1 between slices. Then, Pearson's correlation coefficients were calculated between each white-matter voxel and subsampled node, resulting in a 18,018 × 4,481 correlation matrix for each subject. To obtained the averaged correlation matrices for subsequent clustering, the individual correlation matrix was first averaged across healthy control (HC) group and antipsychotic-naive first-episode schizophrenia (FES) group separately, and then averaged again across the two groups. Next, K-means clustering (distance metric-correlation, 10 replicates) was employed on the averaged correlation matrices. The numbers of clusters ranging from 2 to 22 were measured to obtain the most stable number of networks. The stability of the number of clusters was assessed by an averaged Dice's coefficient (the threshold was set at 0.85) . Specifically, for each number of clusters, the whole connectivity matrix was randomly divided into four folds, and each fold was then clustered identically. The similarity between the clustering in different folds was measured by comparing its adjacency matrix using Dice's coefficient. Finally, the most stable and detailed white-matter functional network masks was obtained for further analyses.

**Figure S1. Stability of clustering for different numbers of clusters.** This graph provides the Dice's coefficient for each number of clusters, and shows that the most stable segregation number was eleven.

**Supplementary 2.**

**Coefficient Granger causality analyses**

Coefficient Granger causality analysis (cGCA) is based on the multivariate autoregressive model including all measured variables as follows:


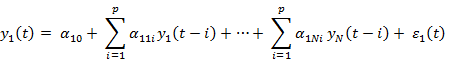


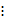


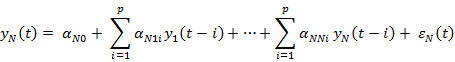


In this model,
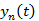
represents averaged time course of each network (n = 1,2,…,N). *p* is the order of the autoregressive model, which was set to 1 using the Schwarz criterion in this study.
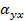
denotes the contribution of source variable x to the prediction of its target y.
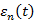
denotes the prediction error (n = 1,2,…,N).

**Disturbance of correlation analyses**

For diluting the effect of direct correlation, Pearson's correlation coefficients among white-matter functional networks were calculated and transformed to Fisher z scores. Then the z score of each edge was added as a covariate in between-group comparison of cGCA using a multiple regression model.

Consistent statistical results were observed (P < 0.05, network-based statistic (NBS) corrected) after regressing the direct correlation. Specifically, decreased excitatory influences from the middle network to the superficial network (t = –3.88, P = 0.0004) and the deep network (t = –3.52, P = 0.0005), as well as decreased inhibitory influences from the fronto-parietal network to the pre/post-central network (t = –2.95, P = 0.005), temporofrontal network (t = –2.97, P = 0.003), and the orbitofrontal network (t = –2.91, P = 0.004) were observed in FESs compared with HCs. The results indicated that the causality we observed was less affected by the disturbance of correlation.

**Supplementary 3.**

Two relative stricter group-level masks (percentages >70% and 80%) were applied to identify white-matter voxels, and the white-matter network clustering and Granger causality analyses were performed again using these stricter masks.

The clustering analyses were similar to the study by Jiang and colleagues and is briefly described here. Specifically, the individual white-matter, gray-matter and CSF masks, which were obtained by using the T1-weighted anatomical image segmentation results, were averaged across all the subjects. As a consequence, the percentage of subjects that were classified as white-matter were obtained. Here, the percentage was set 70% or 80% respectively to eliminate the potential interference from neighboring gray-matter. Finally, the subcortical areas were removed from the white-matter mask. Using the two stricter group-level masks, the clustering analyses were then performed. Table S1 shows that the group-level white-matter masks (percentages > 70% and > 80%) had less voxels than the masks (percentage > 60%) in current and previous studies .

The Granger causality analyses were performed again to obtained influence coefficient matrices under the two stricter group-level masks respectively. Correlation analyses were computed between any two influence coefficient matrices to evaluate their similarities (Table S2). Moreover, within-group networks influence patterns and between-group networks influence differences were calculated again using results from the two stricter masks respectively (Figure S2). All together, the high correlation coefficient and the consistent statistical results under different masks suggested that the main results were still stable even with stricter masks.

**Table S1. The voxel number within group-level white-matter mask.**

| Peer's study | Luo's study | The current study | | |
| --- | --- | --- | --- | --- |
| 60% mask | 60% mask | 60% mask | 70% mask | 80% mask |
| 20,059 | 18,591 | 18,018 | 16,759 | 15,546 |

**Table S2.** **The correlation coefficient between any two white-matter influence coefficient matrices generated from different masks.**

| correlation coefficient | | Influence coefficient matrix | | |
| --- | --- | --- | --- | --- |
|  |  | Mask, 60% | Mask, 70% | Mask, 80% |
| Influence coefficient matrix | Mask, 60% | - | 0.9956 | 0.9856 |
|  | Mask, 70% | 0.9956 | - | 0.9956 |
|  | Mask, 80% | 0.9856 | 0.9956 | - |


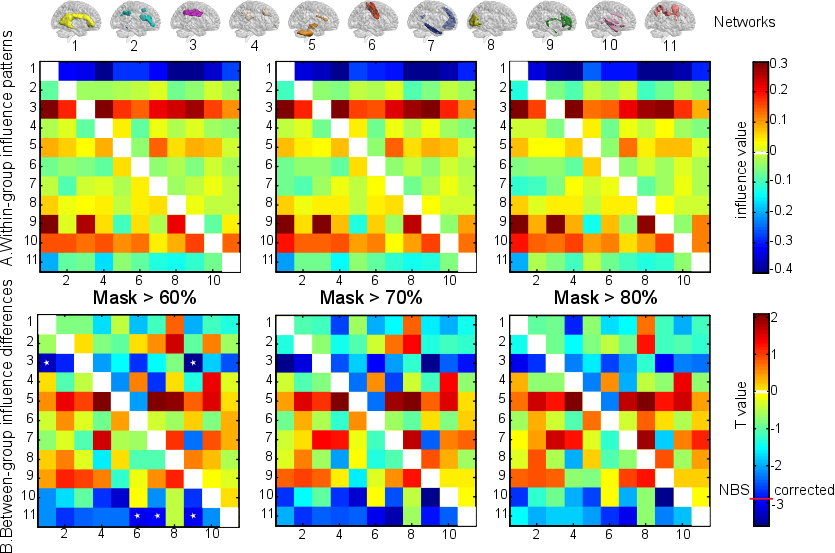


**Figure S2.** **White-matter network influence generated from three masks (threshold > 60%, 70% and 80%).** (A) Average network influence coefficient patterns across all participants. (B) Network influence differences between groups (antipsychotic-naive first-episode schizophrenia patients vs. healthy controls). Color bar shows the t value from the two-sample t-test with 5000 permutations (P <0.05, NBS corrected). NBS, network-based statistic. The color bar shows the t value from the two-sample t-test with 5000 permutations (P <0.05, NBS corrected). NBS, network-based statistic.

**Supplementary 4.**

Differences on 11 networks influence between FESs and HCs were measured using two-sample t-tests with 5000 permutations (*P* < 0.05, NBS corrected; Figure S3). Compared with HCs, FESs exhibited decreased excitatory influences from the superior corona radiate network to the orbitofrontal network (T = -3.62, P < 0.001), as well as the deep network (T = -3.25, P = 0.001) Moreover, FESs exhibited decreased inhibitory influences from the permotor/posterior parietal network to the pre/post-central network (T = -2.94, P = 0.004), tempofrontal network (T = -2.98, P = 0.002), and the orbitofrontal network (T = -3.04, P = 0.001).


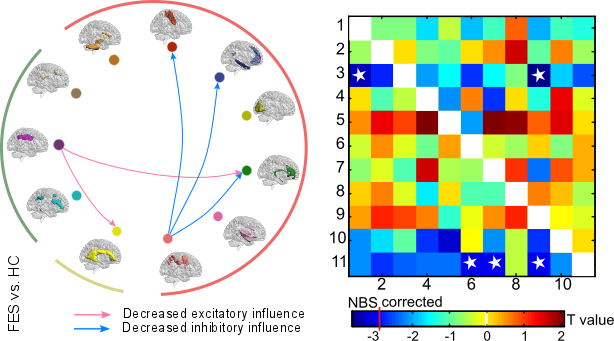


**Figure S3. Between-group differences of influence among 11 white-matter functional networks.** The color bar shows the t value from the two-sample t-test with 5000 permutations. ★ denotes P <0.05, NBS corrected. NBS, network-based statistic; FES, antipsychotic-naive first-episode schizophrenia patient; HC, healthy control.

**Supplementary 5.**

**Data acquisition.** Scans were acquired on a 3.0 Tesla MRI scanner (GE Medical Systems, Waukesha, WI, USA) at the First Affiliated Hospital of Chongqing Medical University. Participants were instructed to stay awake with their eyes closed and not to think of anything in particular. T1-weighted anatomical images were acquired by a three-dimensional fast spoiled gradient-echo sequence with the following parameters: TR = 835 ms; TE = 3.27 ms; flip angle = 120°; field of view = 250 × 250 mm^2^; matrix = 256 × 256, 156 axial slices; slice thickness = 1 mm, no gap. Resting-state functional images were acquired using an echo-planar imaging (EPI) sequence with the following parameters: TR = 2000 ms; TE = 30 ms; flip angle = 90°; field of view = 240 × 240 mm^2^; matrix = 64 × 64, 33 axial slices; slice thickness = 4 mm, 0.6mm gap; 240 volumes.

**Table S3. Demographic and Clinical Characteristics.**

| Characteristic | HCs  (n = 12) | FESs  (n = 12) | Group comparisons | |
| --- | --- | --- | --- | --- |
|  |  |  | Statistic values | P values |
| Sex (male/ female) | 7/ 5 | 8/ 4 | χ^2^ = 0.18 ^a^ | .67 |
| Age (years) | 28.42 ± 3.03 | 28.08 ± 3.72 | U = 66.50 ^B^ | .76 |
| Education (years) | 12.08 ± 1.14 | 10.17 ± 0.84 | U = 51.00 ^B^ | .22 |
| Cigarette use (no/ yes) | 7/ 5 | 6/ 6 | χ^2^ = 0.17 ^a^ | .68 |
| Alcohol use (no/ yes) | 12/ 0 | 11/ 1 | χ^2^ = 1.04 ^a^ | .31 |

mean ± SEM; ^a^ The χ^2^ value for gender distribution was obtained by chi-square test; ^b^ The U values were obtained by Mann-Whitney tests; HC, healthy controls; FES, antipsychotic-naive first-episode schizophrenia patients.


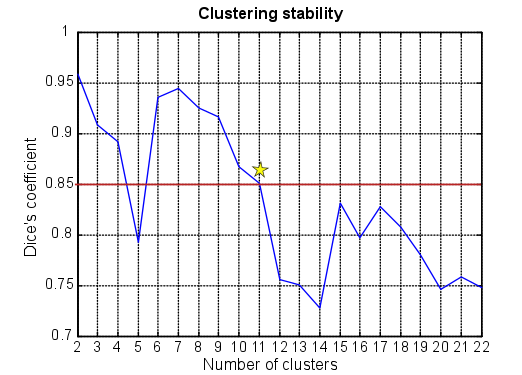


**Figure S4. Stability of clustering for different numbers of clusters in the replication cohort.** This graph provides the Dice's coefficient for each number of clusters, and shows that the most stable segregation number was eleven.

**References**

Buckner, R. L., Krienen, F. M., Castellanos, A., Diaz, J. C., & Yeo, B. T. (2011). The organization of the human cerebellum estimated by intrinsic functional connectivity. *J Neurophysiol, 106*(5), 2322-2345. doi: 10.1152/jn.00339.2011

Craddock, R. C., James, G. A., Holtzheimer, P. E., 3rd, Hu, X. P., & Mayberg, H. S. (2012). A whole brain fMRI atlas generated via spatially constrained spectral clustering. *Hum Brain Mapp, 33*(8), 1914-1928. doi: 10.1002/hbm.21333

Desikan, R. S., Segonne, F., Fischl, B., Quinn, B. T., Dickerson, B. C., Blacker, D., . . . Killiany, R. J. (2006). An automated labeling system for subdividing the human cerebral cortex on MRI scans into gyral based regions of interest. *Neuroimage, 31*(3), 968-980. doi: 10.1016/j.neuroimage.2006.01.021

Jiang, Y., Luo, C., Li, X., Li, Y., Yang, H., Li, J., . . . Yao, D. (2018). White-matter functional networks changes in patients with schizophrenia. *Neuroimage*. doi: 10.1016/j.neuroimage.2018.04.018

Lorio, S., Fresard, S., Adaszewski, S., Kherif, F., Chowdhury, R., Frackowiak, R. S., . . . Draganski, B. (2016). New tissue priors for improved automated classification of subcortical brain structures on MRI. *Neuroimage, 130*, 157-166. doi: 10.1016/j.neuroimage.2016.01.062

Peer, M., Nitzan, M., Bick, A. S., Levin, N., & Arzy, S. (2017). Evidence for Functional Networks within the Human Brain's White Matter. *J Neurosci, 37*(27), 6394-6407. doi: 10.1523/JNEUROSCI.3872-16.2017
